# Diffusion-controlled reaction rates for two active sites on a sphere

**DOI:** 10.1186/2046-1682-7-3

**Published:** 2014-06-04

**Authors:** David E Shoup

**Affiliations:** 1 Mathematics and Science Department, Lincoln Land Community College, 5250 Shepherd Rd, P.O. Box 19256, Springfield, IL 62794, USA

## Abstract

**Background:**

The diffusion-limited reaction rate of a uniform spherical reactant is generalized to anisotropic reactivity. Previous work has shown that the protein model of a uniform sphere is unsatisfactory in many cases. Competition of ligands binding to two active sites, on a spherical enzyme or cell is studied analytically.

**Results:**

The reaction rate constant is given for two sites at opposite ends of the species of interest. This is compared with twice the reaction rate for a single site. It is found that the competition between sites lowers the reaction rate over what is expected for two sites individually. Competition between sites does not show up, until the site half angle is greater than 30 degrees.

**Conclusions:**

Competition between sites is negligible until the site size becomes large. The competitive effect grows as theta becomes large. The maximum effect is given for theta = pi/2.

## Background

**Figure 1:**
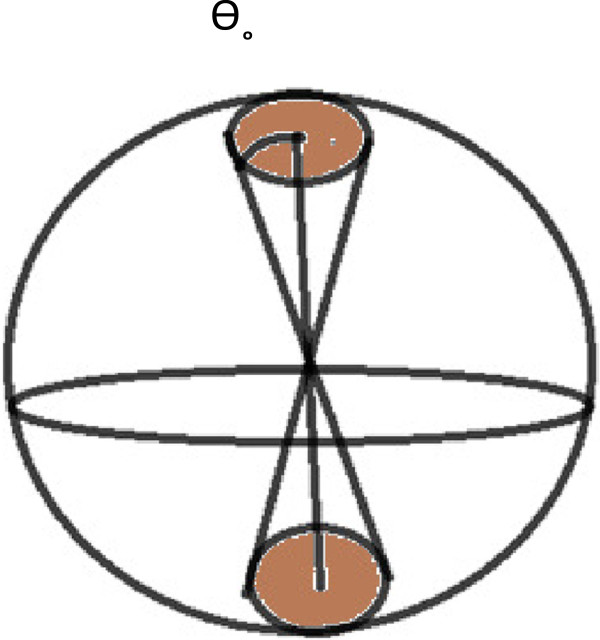
**A non-reactive sphere with two reactive spherical caps of half angle θ**_**° **_**at each end.**

The purpose of this article is to generalize the Smoluchowski [1] calculation of the steady state bimolecular rate constant, 4πDR, to anisotropic reactivity. This was necessary because previous work [2,3], has shown that diffusion-controlled protein-ligand binding is not modeled successfully by a uniformly reactive protein for some cases. As a consequence of this, more realistic models of proteins were created. Previously this was done by having one reactive site on a spherical molecule or cell [4-6]. Berg and Purcell considered many sites on a sphere [7]. This paper considers two axially symmetric sites on opposite ends of a sphere reacting with small molecules. See Figure 1. This might be used to model a protein-ligand reaction: such as the diffusion-controlled reaction of acetylcholine with the active sites of tetrameric mouse acetylcholinesterase [8].

## Methods

We want to solve

(1) $$\begin{array}{l} \partial^{2}c\left(r,\theta \right)/\partial r^{2}+\left(2/r\right)\partial c\left(r,\theta \right)/\partial r\\ \;+1/r^{2}\left(1/\text{sin}\;\theta \right)\partial /\partial \theta \left(\text{sin}\;\theta \partial c\;\left(r,\theta \right)/\partial \theta \right)\\ \;=0 \end{array}$$

where c(r, θ) gives the ligand concentration. Figure 1 shows that there is symmetry between the two active sites. As a consequence of this, the net flux normal to the equatorial plane vanishes for all r. This reduces the two site problem to a one site problem, where now θ varies from 0 to π/2. Mathematically this says

(2) $$\partial c\left(r,\theta \right)/\partial \theta =0\;\theta =\pi /2$$

We also have

(3) $$c_{{}_{{}^{\circ}}}={\text{lim}}_{r\to \infty }c\left(r,\theta \right)$$

where c˳ is the bulk concentration of ligand. The reactive boundary conditions are given by

(4) $$c\left(R,\theta \right)=0\;0\leq \theta \leq \theta_{\circ }$$

and

(5) $$\partial c\left(r,\theta \right)/\partial r|{}_{r=R}=0\;\theta_{{}^{\circ}}\leq \theta \leq \pi /2$$

As done previously [6,9], equation (4) is replaced by the constant flux boundary condition

(6) $$\partial c\left(r,\theta \right)/\partial r|{}_{r=R}=Q\;0\leq \theta \leq \theta_{\circ }$$

where Q is evaluated, by requiring the average concentration of ligand to vanish over the active site. (see ref. [6], equation 8, κ → ∞)

(7) $$\int_{0}^{\theta ˳}c\left(R,\theta \right)\text{sin}\theta d\theta =0$$

The diffusion limited rate constant for the two site problem is

(8) $$k_{\mathrm{DC}}=\frac{2\pi R^{2}D}{c_{\circ }}\int_{0}^{\theta_{\circ }}\frac{\partial c}{\partial r}|{}_{r=R}\text{sin}\theta d\theta$$

Where D is the diffusion coefficient of the ligands and R is the radius of the sphere. The accuracy of the constant flux boundary condition may be seen, for small θ˳ (e.g. small binding sites), by considering a reactive disk in an insulating plane. For this problem, the exact solution is known [6]. It is k_DC_ = 4 Da, where a = the disk radius. The constant flux method yields [6] k_DC_ = 3.7 Da. Thus the constant flux boundary condition is accurate for small reactive sites.

The solution of equation (1) is given by

(9) $$c\left(r,\theta \right)=\alpha +\sum_{m}^{\infty }a_{m}f_{m}\left(r\right)P_{m}\left(\text{cos}\theta \right)$$

where the *P*_*m*_ (x) are Legendre polynomials of order m. Application of the boundary condition given by equation (2) yields

(10) $$c\left(r,\theta \right)=\alpha +\sum_{m}^{\infty }\alpha_{2m}f_{2m}\left(r\right)P_{2m}\left(\text{cos}\theta \right)$$

Equation (3) is satisfied with α = c˳ and with the radial functions

(11) $$f_{2m}\left(r\right)=1/r^{2m+1}$$

The coefficients a_2m_ in equation (10) are found using equations (5) and (6). Details, parallel a previous derivation [6].

## Results and discussion

In this section we present the results and discuss how the method represents a step forward in the field of diffusion-controlled kinetics and biophysics in general.

The results are given by the solution of the model in the preceding section, and by comparing it to twice the rate limited constant for the one site problem [6]. The difference between the two problems, gives a measure of competition between the two sites for reaction with ligands.

The diffusion limited rate constant for the two site problem is

(12) $$k2=k_{\mathrm{DC}}/4\pi \mathrm{DR}$$

where 4πDR is the rate constant for a uniformly reacting sphere. The reciprocal of our rate constant is

(13) $$\begin{array}{l} 1/k2=1+\frac{1}{4{\left(1-\text{cos}\theta \circ \right)}^{2}}\;\sum_{m=1}^{\infty }\\ \;\left({\left(P_{2m-1}\left(\text{cos}\theta \circ \right)-P_{2m+1}\left(\text{cos}\theta \circ \right)\right)}^{2}/\left(m+\frac{1}{2}\right)(2m+\frac{1}{2}\right) \end{array}$$

The reciprocal of the rate constant for one site is given by

(14) $$\begin{array}{l} 1/k1=1+\frac{1}{2{\left(1-\text{cos}\theta \circ \right)}^{2}}\;\sum_{m=1}^{\infty }\\ \;\left({\left(P_{m-1}\left(\text{cos}\theta \circ \right)-P_{m+1}\left(\text{cos}\theta \circ \right)\right)}^{2}/\left(m+\frac{1}{2}\right)(m+1\right) \end{array}$$

**Figure 2:**
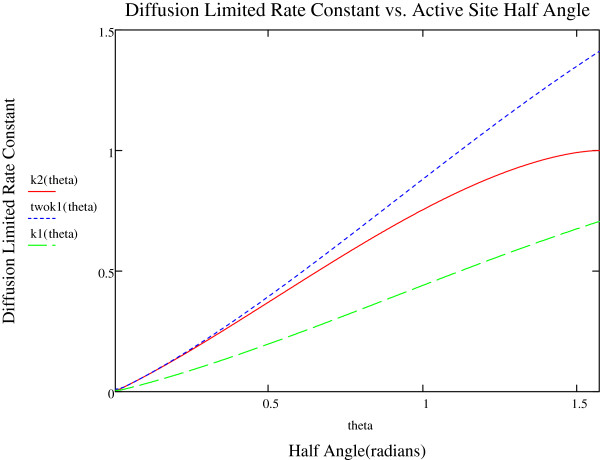
Plot showing the variation of the diffusion-limited rate constant with half-angle theta for 3 different cases.

Figure 2 shows plots of *k2* (θ˳) (the rate constant for 2 sites), twok1 (θ˳) (twice the rate constant for 1 site) *and k*1 (θ˳) (the rate constant for one site [6]) versus θ_°_. For small θ˳, the two site rate constant and 2 × the single site rate constant are in agreement with each other, as would be expected(both behaving as two active sites on a large sphere). As θ˳ grows above 30 degrees, the curves grow apart. The two site curve, being less than 2 × the one site curve.

The difference between the curves is a measure of the competition between the two sites. The competition effect does not show up till around 30 degrees. For $\theta \circ =\frac{\pi }{2},k2=1$, which is the exact result. Thus, the constant flux boundary condition, is good for large θ˳.

This paper represents progress in the field, by presenting a new model for the interpretation of experimental data. This is for macromolecule-ligand binding reactions that fall in the diffusion-controlled regime. Previously, only one site models were available for modeling proteins. Proteins with multiple binding sites can now be studied.

## Conclusions

The analytical expression for the diffusion-limited rate constant to two active sites on a sphere has been given. The result was used to study the competitive effects between the two sites. The effect doesn’t show up until the site half-angles is greater than 30 degrees. The competitive effect grows until its maximum value is reached at θ˳ = π/2.

## Competing interests

There are no competing interests for this paper.

## References

[B1] Smoluchowski MV Versuch einer mathematischen Theorie der Koagulationskinetik kolloider Losungen Z Phys Chem 1917 92 129 168

[B2] Hasinoff BB The diffusion-controlled reaction kinetics of the binding of CO and O_2_ to myoglobin in glycerol-water mixtures of high viscosity Arch Biochem Biophys 1977 183 176 188 10.1016/0003-9861(77)90432-5 907349

[B3] Nakatani H Dunford HB Meaning of diffusion-controlled association rate constants in enzymology J Phys Chem 1979 83,20 2662 2665

[B4] Solc K Stockmayer WH Kinetics of diffusion-controlled reactions between chemically asymmetric molecules. ll approximate steady-state solution In J Chem Kinet 1973 5 733 752 10.1002/kin.550050503

[B5] Samson R Deutch JM Diffusion-controlled reaction rate to a buried active site J Chem Phys 1978 68 285 290 10.1063/1.435494

[B6] Shoup D Lipari G Szabo A Diffusion-controlled bimolecular reaction rates, the effect of rotational diffusion and orientation constraints Biophys J 1981 36 697 714 10.1016/S0006-3495(81)84759-5 7326330 PMC1327653

[B7] Berg HC Purcell EM Physics of chemoreception Biophys J 1977 20 193 219 10.1016/S0006-3495(77)85544-6 911982 PMC1473391

[B8] Zhang D Suen J Zhang Y Song Y Radic Z Taylor P Holst MJ Bajaj C Baker NJ McCammon JA Tetrameric mouse acetylcholinesterase: continum diffusion rate calculations by solving the steady-state smoluchowski equation using finite element methods Biophys J 2005 88 1659 1665 10.1529/biophysj.104.053850 15626705 PMC1305222

[B9] Zhou HX Brownian dynamics study of the influences of electrostatic interaction and diffusion on protein-protein association kinetics Biophys J 1993 64 1711 1726 10.1016/S0006-3495(93)81543-1 8396447 PMC1262506

